# Neutrophil extracellular trap formation and nuclease activity in septic patients

**DOI:** 10.1186/s12871-019-0911-7

**Published:** 2020-01-13

**Authors:** Linda E. Cox, Kai Walstein, Lena Völlger, Friederike Reuner, Alexandra Bick, Annika Dötsch, Andrea Engler, Jürgen Peters, Maren von Köckritz-Blickwede, Simon T. Schäfer

**Affiliations:** 1 Klinik für Anästhesiologie und Intensivmedizin, Universität Duisburg-Essen & Universitätsklinikum Essen, Hufelandstraße 55, D-45122 Essen, Germany; 2 Institut für Physiologische Chemie, Stiftung Tierärztliche Hochschule Hannover, Bünteweg 2, D-30559 Hannover, Germany; 3 Research Center for Emerging Infections and Zoonoses, Stiftung Tierärztliche Hochschule Hannover, Hannover, Germany; 4 Klinik für Anaesthesiologie, Ludwig-Maximilians-Universität München, Munich, Germany

**Keywords:** Neutrophil extracellular traps, Sepsis, Nuclease activity, Mitochondrial DNA

## Abstract

**Background:**

There is little knowledge, whether in patients with sepsis neutrophil extracellular trap (NET) formation and NET degrading nuclease activity are altered. Thus, we tested the hypotheses that 1) NET formation from neutrophils of septic patients is increased compared to healthy volunteers, both without stimulation and following incubation with mitochondrial DNA (mtDNA), a damage-associated molecular pattern, or phorbol 12-myristate 13-acetate (PMA; positive control) and 2) that serum nuclease activities are increased as well.

**Methods:**

Following ethic committee approval, we included 18 septic patients and 27 volunteers in this prospective observational trial. Blood was withdrawn and NET formation from neutrophils was analyzed in vitro without stimulation and following incubation with mtDNA (10 μg/well) or PMA (25 nmol). Furthermore, serum nuclease activity was assessed using gel electrophoresis.

**Results:**

In contrast to our hypothesis, in septic patients, unstimulated NET release from neutrophils was decreased by 46.3% (4.3% ± 1.8 SD vs. 8.2% ± 2.9, *p* ≤ 0.0001) and 48.1% (4.9% ± 2.5 vs. 9.4% ± 5.2, *p* = 0.002) after 2 and 4 h compared to volunteers. mtDNA further decreased NET formation in neutrophils from septic patients (4.7% ± 1.2 to 2.8% ± 0,8; *p* = 0.03), but did not alter NET formation in neutrophils from volunteers. Of note, using PMA, as positive control, we ensured that neutrophils were still able to form NETs, with NET formation increasing to 73.2% (±29.6) in septic patients and 91.7% (±7.1) in volunteers (*p* = 0.22). Additionally, we show that serum nuclease activity (range: 0–6) was decreased in septic patients by 39.6% (3 ± 2 vs 5 ± 0, median and ICR, *p* = 0.0001) compared to volunteers.

**Conclusions:**

Unstimulated NET formation and nuclease activity are decreased in septic patients. mtDNA can further reduce NET formation in sepsis. Thus, neutrophils from septic patients show decreased NET formation in vitro despite diminished nuclease activity in vivo.

**Trial registration:**

DRKS00007694, german clinical trials database (DRKS). Retrospectively registered 06.02.2015.

## Background

Neutrophils play a key role in the response to infection [1–3]. In addition to phagocytosis and intracellular killing of pathogens, they have been shown to actively release extracellular netlike structures (“NET-osis”) that consist of a nuclear DNA backbone, histones, and granular proteins, that can entrap, immobilize, and even kill gram-positive and gram-negative bacteria, fungi, and parasites [4–6].

However, the regulation, if any, of the amount of circulating NETs is largely unknown. In mice with severe sepsis, depending on proinflammatory pathways NETs can be released [7], especially during the initial proinflammatory phase [8], and then degraded by serum nucleases [4, 8]. The main function of human nucleases was said to be the destruction of extrinsic DNA, as ingested via the intestines. However, another important role of nucleases might be the decrease and thus the counterregulation of excessive NET concentration, i.e., to protect the body against negative effects of extracellular traps [8–10]. Accordingly, one might speculate that increased NET formation is associated with increased serum nuclease activity.

Some prior studies investigating NETs in human sepsis determined circulating free DNA (cfDNA) blood concentrations rather than actual NET formation itself [10, 11]. This methodological approach is questionable as cfDNA also includes other non-NET-related types of human DNA, like genomic or mitochondrial DNA (mtDNA) [12, 13]. Thus, all types of circulating DNAs contributed to the measured amount of cfDNA in these studies, which then was found to be increased in septic patients [10, 11] or mice [8]. mtDNA, a damage-associated molecular pattern (DAMP), is increased in patients with severe trauma and activates neutrophils [14, 15]. As mtDNA impacts on immune pathways, it even might influence NET formation. Additionally, phorbol 12-myristate 13-acetate (PMA) an artificial and maximal NET stimulator is widely used as positive control to ensure proper viability of neutrophils [4].

Accordingly, we tested the hypotheses that 1) NET formation from neutrophils of septic patients is increased compared to healthy volunteers, both without stimulation and following incubation with mtDNA or phorbol 12-myristate 13-acetate (PMA; positive control) and 2) that serum nuclease activity is increased as well.

## Material and methods

### Patients’ and volunteers’ characteristics

Following local ethics committee approval (no. 09–4154) and study registration (German clinical trials database, DRKS no. 00007694), we included 18 consecutive septic patients admitted to our intensive care unit (ICU), as well as 27 healthy volunteers, mostly hospital staff, to this prospective, observational trial. Septic patients were eligible if they fulfilled the criteria of sepsis according to the Surviving Sepsis Campaign Guidelines [16]. The SOFA Score of the patients with sepsis averaged 14 ± 2 (mean ± standard deviation), which is accompanied by a calculated 89,7% mortality [17]. All patients needed mechanical ventilation and vasoactive support, and 17 of 18 patients needed more than 0,1 μg/kg/min norepinephrine alone or in combination with dopamine. 9 of 18 patients received haemodialysis. 8 of 18 Patients with sepsis died in the first 30 days after blood sampling (i.e., a 44% mortality). Volunteers were eligible if they did not suffer from any acute or chronic disease, had no vaccination within 14 days prior to blood withdrawal, and were not taking chronic medications (except for oral contraception pills in women). To exclude that volunteers had an unrecognized infection, white blood count and C-reactive protein concentrations were measured and found to be within the normal reference range. Sample size was calculated based on preliminary experiments using an a priori power analysis using G-Power software (G*Power 3.2; Düsseldorf, Germany). An a priori α-error p of 0.05 and a given power of 1-β of 0.95 revealed a sample size of 18 individuals per group, based on the determined effect size from preliminary results. Patients’ and volunteers’ characteristics are presented in Table 1.

**Table 1:** Infection related characteristics of patients and volunteers

Variable	Healthy volunteers (*n* = 27)	Patients with sepsis (*n* = 18)	*p*-value
Mean age, years	33 ± 14	49 ± 17	< 0.0001
Females / males, N (%)	20 (74) / 7 (26)	2 (11) / 16 (89)	< 0.0001
Leukocyte blood concentration (10^3^ μl^− 1^)	6 ± 1	18 ± 15	0.002
Neutrophil blood concentration (10^3^ μl^-1)^	3 ± 0.5	17 ± 7	≤0.0001
Immature neutrophils (% of all neutrophils)	n/a	10 ± 12	
C-reactive protein serum concentration (mg l^− 1^)	≤0.5 ± 0	22 ± 13	≤0.0001
Procalcitonin serum concentration (ng ml^− 1^)	n/a	56 ± 96	
SAPS II	n/a	34 ± 10	
SOFA	n/a	14 ± 2	
30-day survival (%)	100	56	
Gram-positive sepsis N (%)	n/a	6 (33)	
Gram-negative sepsis N (%)	n/a	5 (28)	
Mixed bacterial sepsis N (%)	n/a	3 (17)	
Viral sepsis N (%)	n/a	0	
Mycotic sepsis N (%)	n/a	2 (11)	
Sepsis without microbial detection N (%)	n/a	2 (11)	
Source of infection 1) Pneumonia, N	n/a	8	
2) Acute respiratory distress syndrome (ARDS) subsequent to prior Pneumonia, but only microbial detection in blood, N	n/a	4	
3) Urinary tract infection, N	n/a	1	
4) Abdominal infection	n/a	2	
5) Thoracic infection	n/a	1	
6) No focus detected	n/a	2	

Data from 27 healthy volunteers and 18 septic patients. Data are presented as numbers, percentages, or means ± standard deviation.

*p*-values relate to Student’s t-test for unpaired samples, where applicable.

### Procedures and measurements

Within 24 h after first diagnosing sepsis blood was withdrawn for in vitro experiments and measurements, neutrophils were isolated (see below), and NET-formation assays were performed immediately, both in septic patients (*n* = 18) and healthy volunteers (*n* = 27). For the measurement of serum nuclease activity blood was stored on ice upon withdrawal, centrifuged (2000 g for 10 min), and serum was frozen at − 80 °C until analysis.

### Isolation of neutrophils

Primary blood-derived neutrophils were isolated from fresh blood by density gradient centrifugation using Polymorphprep™ (Progen Biotechnik, Heidelberg, Germany), as described previously [18]. For in vitro NET formation assays, the neutrophils were seeded on poly-L-lysine-coated glass slides in 24-well plates at a concentration of 5 × 10^5^ cells per well (250 μl) in RPMI 1640 medium (Thermo Fisher Scientific Inc., Waltham, MA) at 37 °C and 5% CO_2_, and NET formation was analyzed after incubation for 2 and 4 h, respectively.

### Isolation of mitochondrial DNA

To harvest a high amount of pure mtDNA, the cultivated human cell line HepG2 was used, as described previously [13]. The cultivation took place in RPMI medium mixed with 10% fetal calf serum (FKS) and 1 mM sodium pyruvate (Thermo Fisher Scientific Inc., Waltham, MA) and cells were stored at 37 °C and 5% CO_2_. For the isolation of mitochondria, the Mitochondria Isolation Kit for Cultured Cells (Thermo Fisher Scientific Inc., Waltham, MA) was used according to product description. Purified mitochondria sediments were collected and stored at 4 °C for 24 h before mtDNA was isolated using the DNeasy Blood & Tissue Kit (Qiagen, Hilden, Germany) following the manufacturer’s protocol. The mtDNA concentration was determined photometrically at a wavelength of 280 nm (Biophotometer Plus, Eppendorf, Hamburg, Germany). mtDNA was stored at − 20 °C.

### Assessment and induction of NET formation

NET formation was assessed in septic patients (*n* = 18), and healthy volunteers (*n* = 27) without stimulation (baseline), and following incubation with mtDNA (final concentration: 10 μg/well), Furthermore, phorbol 12-myristate 13-acetate (PMA, 25 nM final concentration), a recognized NET formation inductor [19], was used as positive control, to ensure overall stimulability of neutrophils. In detail, 250 μl RPMI medium including either the respective stimulating agent or vehicle (negative control) was added to 250 μl of cell suspensions (5 × 10^5^ cells/well) in 24 well plates.

To achieve adhesion of neutrophils to the glass-insert all plates were centrifuged at room temperature (22 °C) at 512 g. Incubation time was 2 and 4 h at 37 °C and 5% CO_2_, respectively. Neutrophils and neutrophil derived structures were fixed with 150 μl of 16% paraformaldehyde and the plates were stored at 5 °C until immunostaining.

### Visualization and quantification of NET formation

Since the major backbone of NETs is the DNA, different DNA-intercalating dyes, for example, 4′,6-Diamidino-2-Phenylindole (DAPI), propidium iodide, SYTOX Orange, or SYTOX Green, are widely used to visualize NETs (de Buhr et al., 2016). Importantly, we have recently shown that cationic antimicrobial peptides, for example, the cathelicidin LL-37, which are associated with NETs, block the binding of DNA-intercalating dyes to the NETs and thereby hamper their visualization (Neumann et al., 2014). Therefore, in the present study we here used an antibody-based technique with an antibody directed against histone-DNA complexes, as a typical marker for NETs. NET visualization and quantification was performed as described previously [18], In detail, cell preparations were washed three times with phosphate-buffered saline (PBS), and permeabilized by incubation with 2% bovine serum albumin (BSA) in 0.2% Triton X-100/PBS for 45 min at room temperature. A mouse monoclonal anti DNA/histone H1 complex (mouse IgG2a anti DNA/histone antibody, Merck Millipore, Darmstadt, Germany) was added and cells were incubated overnight at 4 °C. After washing the cells three times with PBS an Alexa-Fluor-488-labelled goat-anti-mouse antibody (Thermo Fisher Scientific Inc., Waltham, MA) was added for 45 min at room temperature. Cells were then washed again and slides mounted in ProlongGold® antifade with DAPI (Invitrogen, Carlsbad, CA) and NET formation was analyzed using fluorescence microscopy (Leica TCS SP5 confocal microscope and Zeiss Anxioveit 200 M non-confocal fluorescence microscope). From each slide three images were randomly selected. Using an antibody against histone-DNA complexes [20], different stages of NET-formation can be identified based on characteristic morphological changes of the nucleus upon stimulation and before release of NETs [18]. During the NET-formation process, disintegration of the nuclear membrane occurs concomitantly with cytoplasmic granule dissolution, allowing NET components to mix in the cytoplasm. The normal lobulated nuclear structure is then broken and a delobulated nuclear form can be found in those cells that are in the early stages of NET-formation. The criteria used for NET-positive cells were: Positively stained green nucleus plus a less dense nucleus (loss of lobulation) or a loss of the round shape of the nucleus plus an increased size of the nucleus, or an occurrence of a distinct extracellular off-shoot [21]. Using this method, the simple counting of dead necrotic cells is excluded based on 1) the antibody staining used and 2) the morphological characteristic of the cells. Data are presented as a percentage of cells showing NET formation related to all neutrophils of an image. For statistical analysis, the mean value of 6 images was used for calculation of average values for each condition and individual.

### Quantification of nuclease activity

Serum nuclease activity of the consecutive septic patients and healthy volunteers was quantified by gel electrophoresis. As negative control tris-(hydroxymethyl)-aminomethan (TRIS)-buffer (300 mM TRIS, 50 mM calcium chloride, 50 mM magnesium chloride) was used. A dilution series of the DNase I (Sigma Aldrich, St. Louis, MO), with an activity range from 2 to 0,0035 units/ml, served as a positive control. TRIS-buffer and calf thymus DNA (Sigma Aldrich, St. Louis, MO) in a concentration of 1 mg/ml was added to the serum samples as well as to the positive and the negative controls. Samples were incubated for 18 h at 37 °C. A phenol chloroform (Carl Roth, Karlsruhe, Germany) extraction was used to separate the DNA from proteins. The resulting fluid phase was mixed with loading buffer (Thermo Fisher Scientific Inc., Waltham, MA) and added to an agarose gel pocket (1%), followed by gel electrophoresis at 100 V for 30 min.

For semiquantification of serum nuclease activity, we compared the gel lane from samples to a dilution series of DNase I. The possible activity range was related to categories 1–6. This grading correlates with DNase I activity of zero (activity range 1), < 0,007 U/ml (range 2), 0,007 U/ml (range 3), 0,007–0,015 U/ml (range 4), 0,015–0,06 U/ml (range 5), and ≥ 0,06 U/ml (range 6), respectively.

### Statistical analysis

Microsoft Excel 2016 (V16, Microsoft, Redmond, WA) and GraphPad Prism (V 6, GraphPad Software, San Diego, CA) were used for data analysis. Data are presented as means (± standard deviation) unless indicated otherwise. The Student’s two-tailed t-test for independent samples or, in case of violation of the normality assumption (as tested by the Kolmogorov-Smirnov and Shapiro-Wilk tests), the Wilcoxon signed rank test was used. Potential associations between serum nuclease activity and clinicopathogenic variables of septic patients like C-reactive protein and procalcitonin serum concentrations were determined using Spearman correlation analysis. Null hypotheses were rejected and statistical significance assumed with an a priori alpha error p of less than 0.05.

## Results

### Unstimulated NET formation

**Fig. 1:**
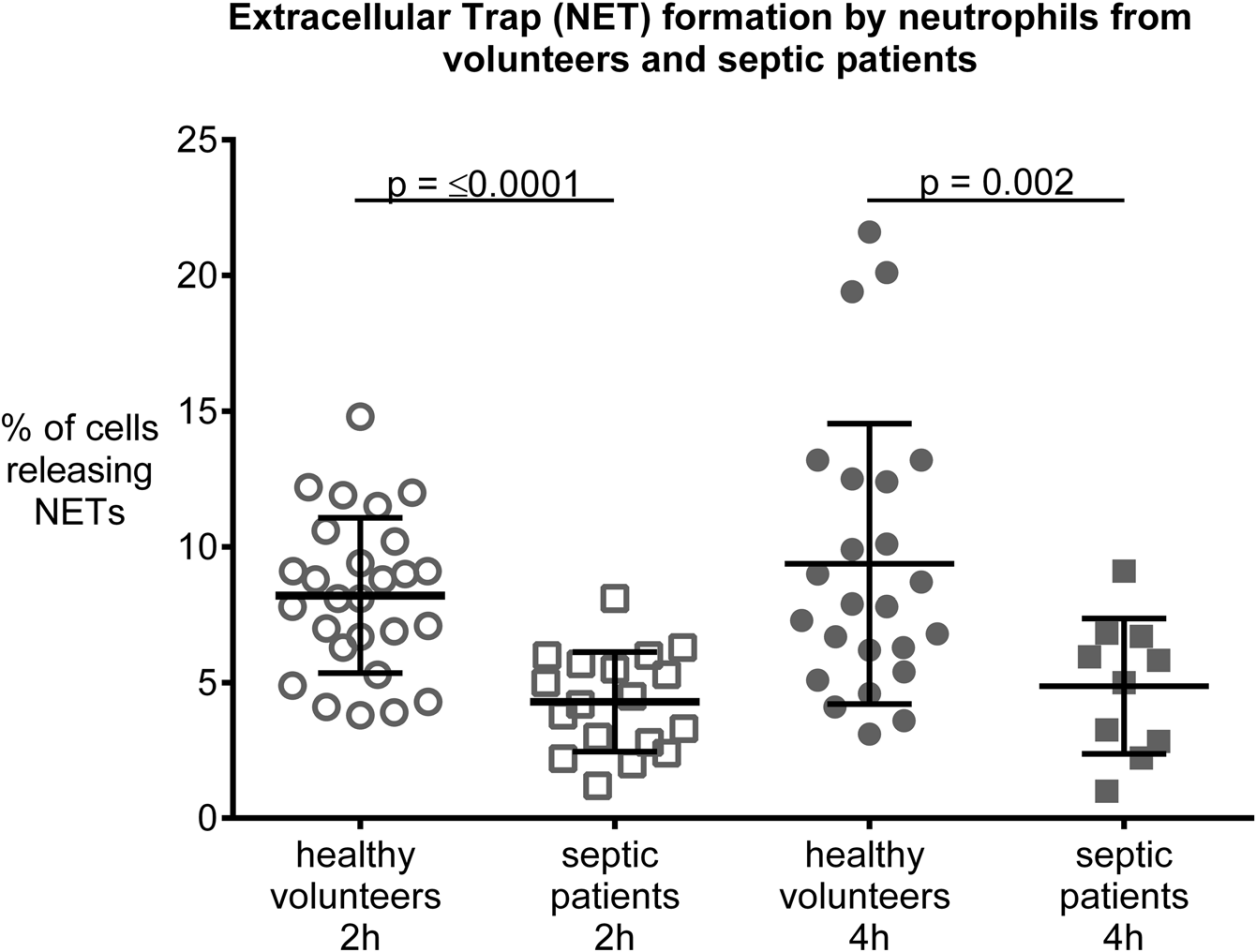
Extracellular Trap (NET) formation by neutrophils obtained from blood of septic patients and volunteers following incubation for 2 and 4 h. Neutrophils from septic patients released significantly less NETs than those from volunteers under baseline conditions both after incubation for 2 and 4 h, respectively. Data are means ± SD

Unstimulated NET formation from neutrophils of septic patients was significantly lower than in healthy volunteers (all *p* < 0.0001). In detail, after 2 h NET formation in septic patients was decreased by 46.3% (4.3% ± 1.8 vs. 8.2% ± 2.9, *p* < 0.0001) and 48.1% (4.9% ± 2.5 vs. 9.4% ± 5.2, *p* = 0.002) after 4 h, compared to healthy volunteers (Fig. 1).

### NET formation following mtDNA stimulation

**Fig. 2:**
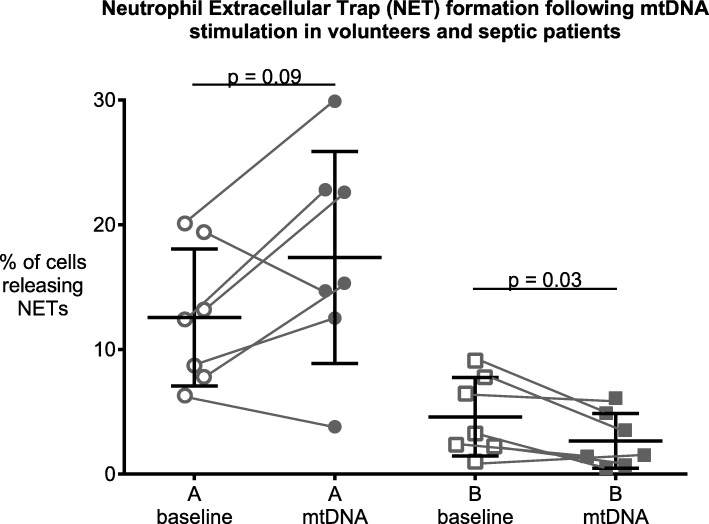
Neutrophil Extracellular Trap (NET) formation in vitro at baseline (open symbols) and after 4 h of incubation with mitochondrial DNA (mtDNA, full symbols) in neutrophils from volunteers (left panel, A) and septic patients (right panel, B). While NET formation in neutrophils from volunteers (A) is unaltered by mtDNA, mtDNA evoked a marginal decrease in NET formation in neutrophils from septic patients (B). Data are individual values and means ± SD

mtDNA decreased NET formation in neutrophils from septic patients (4.7% ± 1.2 to 2.8% ± 0,8; *p* = 0.03, Fig. 2), which was not seen in healthy volunteers (12.6% ± 5.5 vs. 17.4% ± 8.5, *p* = n.s.)

### NET formation following PMA stimulation

**Fig. 3:**
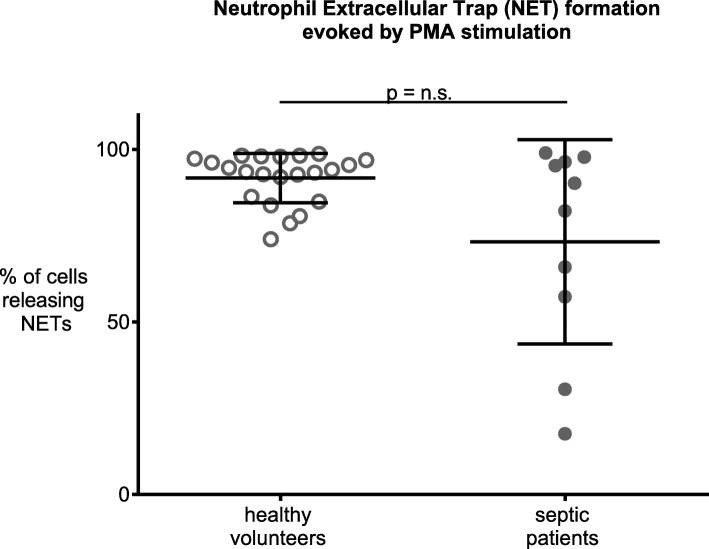
Neutrophil Extracellular Trap (NET) formation following phorbol-myristate-acetate (PMA) stimulation. PMA (25 nM) led to a strong increase of NET formation, both in septic patients and volunteers, reaching NET formation in more than 70% of neutrophils. Thus, in spite of decreased NET formation under baseline conditions in septic patients, NET formation capacity in neutrophils from septic patients is still high and approaches that of PMA stimulated neutrophils from volunteers. Data are means ± SD

Positive control PMA increased NET formation to greater than 70% both in septic patients and healthy volunteers, indicating that neutrophils were vital and capable for NET formation following maximum artificial stimulation. As expected, maximum NET formation did not differ between groups (healthy volunteers: 91.7% ± 7.1 vs. septic patients: 73.2% ± 29.6, *p* = 0.22, Fig. 3).

### Nuclease activity

**Fig. 4:**
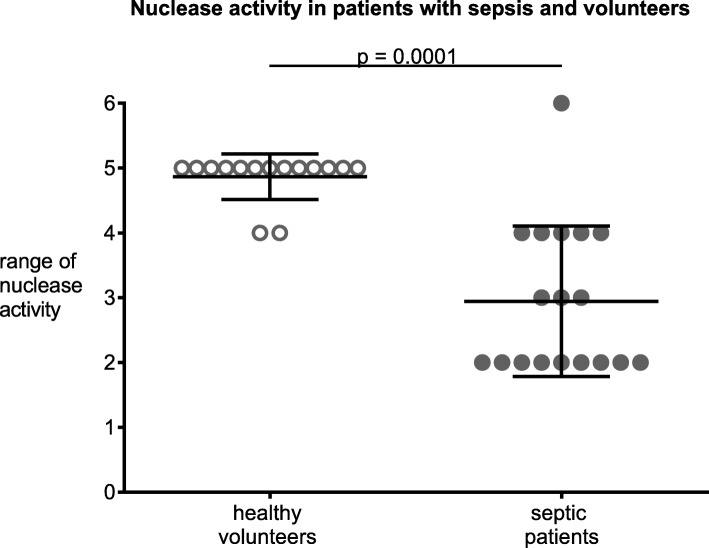
Nuclease activity in serum of patients with sepsis and volunteers. Patients with sepsis show a marked decrease of nuclease activity compared to that of volunteers. Data are median ± interquartile range

Nuclease activity in septic patients was markedly decreased by 39.6% (3 ± 2 vs 5 ± 0; median ± interquartile range; *p* = 0.0001, Fig. 4). Interestingly, C-reactive protein (*r* = − 0.904; *p* = 0.035) and procalcitonin serum concentrations (*r* = − 0.918; *p* = 0.028) showed an inverse correlation with nuclease activity whereas the Simplified Acute Physiology Score II (SAPS II) did not (*r* = − 0.839; *p* = 0.08).

## Discussion

In septic patients 1) unstimulated ex vivo NET formation is decreased, 2) mtDNA further decreases NET formation, and 3) serum nuclease activity is decreased in septic patients compared to healthy volunteers. Thus, our results show, that both NET formation and degradation are diminished in septic patients.

Surprisingly, unstimulated neutrophils from septic patients released less NETs compared to neutrophils from healthy volunteers. Therefore, we need to consider possible confounders. First, could serum proteins in septic patient’s blood block NET formation. This is unlikely as we studied a standardized number of isolated and several times washed neutrophils. Thus, remaining serum is highly low and a depressant effect of serum components on ex vivo NET formation can almost be ruled out. Second, might a methodological aspect have led to decreased NETosis This is unlikely as well, as applying the extremely strong stimulant PMA, which is routinely used as positive control, cells performed maximal NETosis, which did not differ between groups. Thus, it is also unlikely, that the intracellular machinery for NET formation was dysfunctional in neutrophils from septic patients. By the same token, it is also unlikely that neutrophils isolated from septic patients were “exhausted” or still more “juvenile” than those from healthy volunteers since both groups had equally high PMA evoked NET formation.

Even more important we need to discuss our findings in context with recent publications. Some prior studies already focused on NET formation in sepsis [10, 11]. However, these studies did not use the gold standard of visualization and quantification of actual NETosis in vitro. In contrast, they used an easy approach and measured total circulating free DNA (cfDNA). As we and others have shown recently, in sepsis cfDNA includes other non-NET-related types of human DNA, like genomic or mitochondrial DNA (mtDNA) [13, 14]. Furthermore, cfDNA cannot discriminate between DNA motives actively released by neutrophils and increased concentrations i.e. related to cell death [22]. Thus, measuring the amount of total cfDNA does not equal NETosis. In the aforementioned publications, all types of circulating DNAs contribute to the measured amount of cfDNA, which than was found to be increased in septic patients [10, 11] or mice [8]. Another work showed increased concentrations of cfDNA in patients with sepsis despite decreased NET release, which supports our argumentation [23]. Thus, direct quantification of NETs in vivo should be used as methodological gold standard. In this respect, Gavillet et al. recently established a flow cytometric assay to directly visualize NETs [24]. This might be a feasible, less time consuming, and easier method for NET quantification in further studies.

Of note, Hashiba et al. measured both cfDNA and NET formation in septic patients by direct visualization using the approach of Fuchs as published previously [5]. Even, when the method is established, the specific PMA assay used did not induce maximal NETosis, both from neutrophils of septic and nonseptic individuals. This is in contrast to the definition of PMA as positive control for NET formation [4, 5, 25]. Thus, as published previously PMA evokes maximum NET release, as PMA is an extremely strong NET inductor and therefore maximum NETosis is required following PMA stimulation to ensure cell viability prior to the experiment or adequate experimental setup [4, 5, 25]. Interestingly, results from NETosis and cfDNA measurement in this study differed, confirming the finding that cfDNA contains DNA from various sources.

In this regard mtDNA, a danger associated molecular pattern, is of particular importance, as mtDNA is known to have many effects on the immune system. It increases the TNF-α, interleukin-1β, and hypoxia-inducible factor-1α mRNA expression in humans [13]. It has also been shown that mtDNA strongly stimulates polymorphonuclear neutrophils [12]. Therefore, mtDNA, might stimulate NET formation. In contrast to our hypothesis, following mtDNA incubation, NET formation was decreased. However, basal, unstimulated, NET formation in our experiments was already low, so that we could only identify a modest drop in NETosis from neutrophils of septic patients. This is interesting, as Zhang et al. had shown that mitochondrial DAMPS, being a mixture of mtDNA and mitochondrial proteins stimulate neutrophils [12], whereas highly purified mtDNA alone, as we used it in our experiments, did not activate neutrophils [26]. As we and others have shown recently, mtDNA can induce immunosuppressive phenotypes, by inhibiting cytotoxic T-cell activity, both in wildtype mice and humans with sepsis [12, 13]. Since mtDNA serum concentrations are increased in trauma and sepsis [13], and excessive NET formation might endanger the organism [10, 27], one might speculate that the body as a self-protective feature does not form NETs in response to mtDNA.

Another explanation for reduced NET release in sepsis might be an acidotic pH of septic patients, as shown by Patel et al., who measured NET release ex vivo in a cohort of septic patients comparable to our cohort [23]. In fact, we included patients with septic shock, as all of them needed vasoactive medication. The SOFA score in our cohort was 14 ± 2 (mean ± standard deviation) and we were able to show a comparable reduction of NET release. The septic patients we included also had an acidotic acid base status at the time of blood collection. Their pH was 7.3 ± 0.09 and the standardized base excess was − 3 ± 5 (means ± standard deviation). No correlation was seen in our patients between the severity of acidosis and decreased NET release or 30-day mortality. Please note, however, that the assessment of the acid base status might be limited because 50% (9 of 18) of our septic patients received continuous haemodialysis. Finally, we assessed serum nuclease activity, which we found to be decreased in septic patients. The combination of decreased basal NET formation and decreased serum nuclease activity is interesting at it might ensure that decreased basal NET formation is not further diminished by high nuclease serum concentrations. On first sight, our data appear to contrast those of Meng et al., reporting increased DNAse concentrations in septic mice [8]. However, in addition to potential differences between mice and human patients, it needs to be pointed out that we measured the nuclease activity and not the concentration irrespective of activity.

Finally, leukocyte and neutrophil counts differed between septic patients and healthy volunteers, with septic patients having increased blood neutrophil count (17.2 × 10^3^/μl ± 7.7 vs. 3.4 × 10^3^/μl ± 0.6; *p* < 0.0001). Thus, when calculating the product of basal NET release and neutrophil count both in septic patients and healthy volunteers, NET formation capacity would be higher in septic patients.

Thus, combination of decreased nuclease activity and increased neutrophil count might at least restore total in vivo NET formation capacity in septic patients despite decreased NET formation in vitro.

Our study has limitations. First, sepsis is a long lasting disease with an initial proinflammatory phase overlapping with later immunoparalysis [28–30]. Our analysis took place in the early proinflammatory phase, when blood neutrophil concentrations are usually increased [31]. Nevertheless, the decreased NETs release might possibly result from both a negative feedback mechanism as well as an immunosuppressive pattern. As stated above 30-day mortality was 44%, with the time of death following ICU admission ranging from day 1 to 30. In detail, patients died on days 2, 12, 14, 15, 16, 16, 17, and 29. Thus, the time of death occurred at early, intermediate, and late time points. Accordingly, patients might have been in either immunostimulatory and immunosuppressive phases or in between. Furthermore, more and more evidence suggests that there is an early overlap of immunostimulatory and immunosuppressive patterns in sepsis. Hence, we do not know whether NET formation may be different during later phases of sepsis. In septic mice, cfDNA concentrations had decreased 48 h after induction of sepsis when compared to 24 h, while DNAse concentration remained unaltered [8]. Obviously, while allowing in vitro measurements of NET formation in response to mediators in washed neutrophils and normalized to cell number, our experiments may not reflect NET behavior in the blood and tissues where different cytokine and cell interactions likely prevail. Accordingly, more studies are necessary to elucidate potential differences in NET formation in different microenvironments. Neutrophils of elderly human seem to release less NETs than those of young individuals [32]. In our cohort, we included more controls and these were not strictly age and gender matched to the patients with sepsis. While the age range of both groups overlapped healthy controls were younger. However, we feel that this is of limited importance as our participant’s age ranges widely and one cannot allocate individuals to a “young” and “old” group. In fact, elderly human have been defined to be 65 years and older in other studies on NET release [33, 34].

In conclusion, neutrophils isolated from septic patients showed reduced NET formation at baseline compared to volunteers. mtDNA, an endogenous DAMP acting on TLR9 receptors, had a modest if any inhibitory effect on neutrophils from septic patients, and serum nuclease activity in septic patients was decreased. Decreased nuclease activity and increased neutrophil count, thus might be counterregulated by decreased basal NET formation. This may be important for protection of the organism from destructive effects of overwhelming NET release in sepsis.

## Data Availability

The final datasets supporting the conclusions of this article are included within the article and its additional files. The raw data that support the findings of this study are available from the corresponding author, LEC, upon reasonable request.

## Abbreviations

BSA: Bovine serum albumin
cfDNA: Circulating free DNA
DAMP: Damage-associated molecular pattern
DAPI: 4′,6-Diamidino-2-Phenylindole
FKS: Fetal calf serum
ICU: Intensive care unit
mtDNA: Mitochondrial DNA
NET: Neutrophil extracellular trap
PBS: Phosphate-buffered saline
PMA: Phorbol 12-myristate 13-acetate
TRIS-buffer: Tris-(hydroxymethyl)-aminomethan-buffer
